# Kaempferol Mitigates CSE‐Induced Lung Injury and Epithelial Cell Ferroptosis via Modulating Nrf2/NCOA4/GPx4 Axis

**DOI:** 10.1111/jcmm.71010

**Published:** 2025-12-31

**Authors:** Fengri Jin, Yanfei Xing, Songyu Li, Zhennan Yi, Aibin Wu, Xin Li

**Affiliations:** ^1^ Department of Medical Oncology Central People's Hospital of Zhanjiang Zhanjiang Guangdong People's Republic of China; ^2^ Department of Ophthalmology Zhaoqing Medical College Zhaoqing Guangdong People's Republic of China; ^3^ Department of Zhanjiang Institute of Clinical Medicine, Zhanjiang Central Hospital, Guangdong Medical University Zhanjiang People's Republic of China; ^4^ Department of Respiratory Medicine Central People's Hospital of Zhanjiang Zhanjiang Guangdong People's Republic of China

**Keywords:** COPD, ferritinophagy, ferroptosis, flavonoids, kaempferol

## Abstract

Ferroptosis, an iron‐dependent regulated necrosis driven by redox imbalance, plays a critical role in the pathogenesis of chronic obstructive pulmonary disease (COPD). Kaempferol (KF), a bioactive flavonoid from *Polygonati Rhizoma*, exhibits anti‐ferroptotic properties in lipid peroxidation disorders, yet its molecular mechanism against cigarette smoke extract (CSE)‐induced ferroptosis in human bronchial epithelial cells (BEAS‐2B) remains to be fully elucidated. Using in vitro models of CSE‐induced injury, we observed that KF restored cell viability and attenuated cytotoxicity by restoring redox equilibrium—significantly elevating glutathione (GSH) while reducing malondialdehyde (MDA) and labile iron pool (Fe^2+^) levels. Mechanistically, KF suppressed ferritinophagy via nuclear receptor coactivator 4 (NCOA4) inhibition and rescued glutathione peroxidase 4 (GPx4) activity, thereby blocking lipid peroxidation cascades. These effects were mediated through Nrf2‐dependent transcriptional activation, counteracting CSE‐triggered Nrf2 pathway dysregulation. Our findings reveal that KF mitigates COPD progression by coordinately targeting the Nrf2/NCOA4/GPx4 axis to inhibit ferroptosis, providing a novel therapeutic strategy for oxidative stress‐driven pulmonary diseases.

**Abbreviations**
ARE: antioxidant response element
COPD: Chronic obstructive pulmonary disease
CSE: Cigarette smoke exposure
DAMPs: damage‐associated molecular patterns
GPx4: glutathione peroxidase 4
GSH: glutathione
LIP: labile iron pool
MDA: malondialdehyde
NCOA4: nuclear receptor coactivator 4
Nrf2: Nuclear factor erythroid 2–related factor 2
PL‐OHs: phospholipid alcohols
PLOOHs: phospholipid hydroperoxides
PUFAs: polyunsaturated fatty acids
ROS: reactive oxygen species
TF: transferrin
TFR1: ferritin receptor

## 1 Introduction

Chronic obstructive pulmonary disease (COPD) is the fourth leading cause of morbidity and mortality, with over 3 million deaths annually [1, 2]. This progressive disorder is characterised by persistent airway inflammation and emphysema, leading to irreversible expiratory airflow limitation [3, 4]. While oxidative stress and chronic inflammation are central to COPD pathogenesis, cigarette smoke exposure (CSE)—the primary risk factor—induces recurrent oxidative damage and aberrant cell death (e.g., apoptosis, necroptosis and ferroptosis), perpetuating lung injury [3]. Despite advances in understanding these mechanisms, current therapies remain palliative, underscoring the need for novel interventions targeting disease‐driving pathways.

Ferroptosis, a regulated iron‐dependent necrotic cell death process, is distinct from apoptosis and autophagy, and is implicated in chronic diseases, including COPD [1, 5, 6]. Its initiation hinges on iron overload and lipid peroxidation due to redox imbalance [7]. Notably, smokers exhibit elevated iron and ferritin levels in alveolar lavage fluid, linking CSE to NCOA4‐mediated ferritinophagy (a selective autophagy process releasing free iron) and subsequent ferroptosis [8, 9, 10]. Yoshida et al. (2019) [1] further demonstrated that GPx4 deficiency exacerbates smoking‐associated ferroptosis, positioning ferroptosis suppression as a potential therapeutic strategy. Thus, targeting iron homeostasis and lipid peroxidation pathways may counteract CSE‐induced lung damage.

Kaempferol (KF; 3,4′,5,7‐tetrahydroxyflavone) is a flavonol and secondary metabolite found in various edible plants. It has been reported that kaempferol, a folk medicine derived from *Polygonati rhizoma*, exhibits anti‐inflammatory and antioxidant effects in controlling inflammatory respiratory diseases. Previous studies suggest that kaempferol may inhibit airway mucin hypersecretion, a pathological feature associated with COPD progression [11]. Recent research has shown that kaempferol possesses significant inhibitory activity against lipid oxidation‐related diseases such as stroke, Alzheimer's disease, and cancer. In vivo, kaempferol has been demonstrated to activate the Nrf2/SLC7A11/GPx4 signalling pathway to inhibit oxygen–glucose deprivation/reperfusion (OGD/R)‐induced ferroptosis and reverse neuronal damage [12]. However, the specific role of kaempferol in CSE‐induced lung ferroptosis and the underlying mechanisms remain unclear.

In the present study, we investigated whether kaempferol can mitigate CSE‐induced ferroptosis in lung epithelial cells. Specifically, we examined if kaempferol treatment could reverse NCOA4‐mediated ferritinophagy and GPx4‐regulated ferroptosis via modulating Nrf2 in vitro. Furthermore, we aimed to elucidate the molecular mechanisms underlying kaempferol's protective effects, focusing on the Nrf2/NCOA4/GPx4 axis, to alleviate lung injury. Our findings not only elucidate the Nrf2/NCOA4/GPx4 axis as a previously unrecognised mechanism through which kaempferol counteracts smoke‐induced ferroptosis in COPD, but also highlight its potential as a multitarget phytotherapeutic agent for combating oxidative stress‐driven pulmonary diseases.

## 2 Methods

### 2.1 Reagents and Antibodies

Kaempferol (purity: 95.0%) was purchased from Sigma. Ferrostatin‐1 (Fer‐1, GC10380) and RSL3 (GC12431) were purchased from MedChemExpress. The MTS assay kit (G3580) was purchased from Promega. The Reduced Glutathione Colorimetric Assay Kit (GSH, E‐BCK030‐M), Lactate Dehydrogenase Activity Assay Kit (LDH, E‐BC‐K046‐M), Cell Ferrous Iron Colorimetric Assay Kit (E‐BC‐K881‐M), and Malondialdehyde Colorimetric Assay Kit (MDA, E‐BC‐K028‐M) were purchased from Elabscience (Wuhan, China). Most primary antibodies were purchased from Cell Signalling Technology (CST), except where specified. Catalogue numbers for each antibody are as follows: Anti‐NCOA4 (Abcam, ab86707), anti‐Ferritin Heavy Chain (Abcam, ab65080), anti‐LC3B (Abcam, ab192890), anti‐Glutathione Peroxidase 4 (GPX4, Abcam, ab125066), Anti‐NRF2 (Proteintech, 16,396–1‐AP), anti‐caspase3 (Proteintech, 19,777–1‐AP), anti‐cleaved caspase3 (Proteintech, 25,128–1‐AP), anti‐β‐actin (Proteintech, 20,536–1‐AP), Anti‐xCT/SLC7A11 (D2M7A, CTX, 12691 s), Goat Anti‐rabbit IgG (Calbiochem, #401315) and Goat Anti‐mouse IgG (Calbiochem, #401215).

### 2.2 Cigarette Smoke Extract Preparation

Cigarette smoke extract (CSE) was prepared as previously described, with minor modifications. CSE was obtained from Suangxipai cigarettes (Guangdong, China). Approximately 30–50 mL of cigarette smoke was drawn into a syringe and transferred to RPMI‐1640 medium in 15 mL BD Falcon tubes. One cigarette yielded 10 mL of CSE solution. The CSE solution was filtered through a 0.22 μm filter (Merck Millipore, SLGS033SS) to remove insoluble particles, resulting in a 100% CSE solution.

### 2.3 Cell Culture

The human bronchial epithelial cell line BEAS‐2B was obtained from the American Type Culture Collection (ATCC, Manassas, VA, USA). BEAS‐2B cells were cultured in RPMI 1640 medium supplemented with 10% fetal bovine serum (FBS), penicillin (100 units/mL), streptomycin (100 μg/mL) and HEPES (25 mM). The cells were maintained at 37°C in a humidified, water‐jacketed incubator with 5% CO2 and 95% air. BEAS‐2B cells were treated with 2 μM Ferrostatin‐1 (Fer‐1) or 5 μM RSL3 for 24 h, beginning 1 h prior to exposure to 2.0% cigarette smoke extract (CSE).

### 2.4 Cell Viability and Cytotoxicity Measurement

Cell viability was quantified using an MTS assay. BEAS‐2B cells were seeded in a 96‐well microplate at a density of 5000 cells per well. The cells were then incubated in serum‐free RPMI 1640 medium containing various concentrations of kaempferol (0, 1, 2.5, 5, 10, 20, 40 and 80 μM), either alone or in combination with different concentrations of CSE (0%, 1%, 2%, 5%, 10% and 20%) for 24 h. Subsequently, 10 μL of MTS solution was added to each well and incubated for 1 h at 37°C. The absorbance was measured immediately at 490 nm. Cell cytotoxicity was evaluated using an LDH assay kit by measuring the absorbance at 450 nm according to the manufacturer's protocol.

### 2.5 Glutathione, Ferrous Iron, and Malondialdehyde Assay

BEAS‐2B cells were seeded in a 96‐well microplate at a density of 5000 cells per well. After washing with PBS, cells were harvested and centrifuged to collect cell pellets. Levels of glutathione (GSH), malondialdehyde (MDA), and intracellular ferrous iron (Fe^2+^) were quantified using the respective colorimetric assay kits per the manufacturer's instructions.

### 2.6 Measurement of Intracellular Reactive Oxygen Species (ROS)

Intracellular ROS levels were measured using the peroxide‐sensitive fluorescent probe 2′,7′‐dichlorofluorescin diacetate (DCFH‐DA), following the manufacturer's instructions. Briefly, DCFH‐DA was diluted to a concentration of 10 μM in serum‐free medium. BEAS‐2B cells were incubated with the diluted DCFH‐DA for 30 min at 37°C. Fluorescence intensity was then measured by flow cytometry under FITC fluorescence detection conditions.

### 2.7 Measurement of Lipid Peroxidation

BEAS‐2B cells seeded in 60‐mm culture dishes were pretreated with 20 μM kaempferol for 1 h at 37°C and then stimulated with 2% CSE for 16 h in serum‐free RPMI 1640. Lipid peroxidation measurements were assessed using a C11‐BODIPY 581/591 probe (Invitrogen, C10445), as previously described. Briefly, cells were incubated with 10 μM C11‐BODIPY 581/591 in growth medium for 30 min, and fluorescence intensity was measured by flow cytometry under FITC fluorescence detection conditions.

### 2.8 Western Blotting

Total proteins were extracted from BEAS‐2B cells using RIPA buffer and quantified using a BCA protein assay (Beyotime, P0012). Equal amounts of total protein (20 μg per sample) were separated by 10%–15% SDS‐PAGE and transferred to a polyvinylidene difluoride (PVDF) membrane (Millipore, ISEQ00010). Membranes were incubated with specific primary antibodies at 4°C for 24 h, followed by HRP‐conjugated secondary antibody incubation and chemiluminescence detection using the iBright Imaging System.

### 2.9 Statistical Analysis

All experiments were performed in triplicate. Data are presented as the mean ± standard deviation (SD). Statistical analysis was performed using GraphPad Prism 8. An unpaired, two‐tailed Student's t‐test with Bonferroni correction for multiple comparisons or a one‐way analysis of variance (ANOVA) followed by Tukey's HSD test was employed where appropriate. Data were first tested for normality and homogeneity of variances to validate the assumptions of the tests used. A *P*‐value of less than 0.05 was considered statistically significant.

## 3 Results

### 3.1 Kaempferol Enhances Cell Viability and Attenuates Cytotoxicity in CSE‐Exposed BEAS‐2B Cells

**FIGURE 1:**
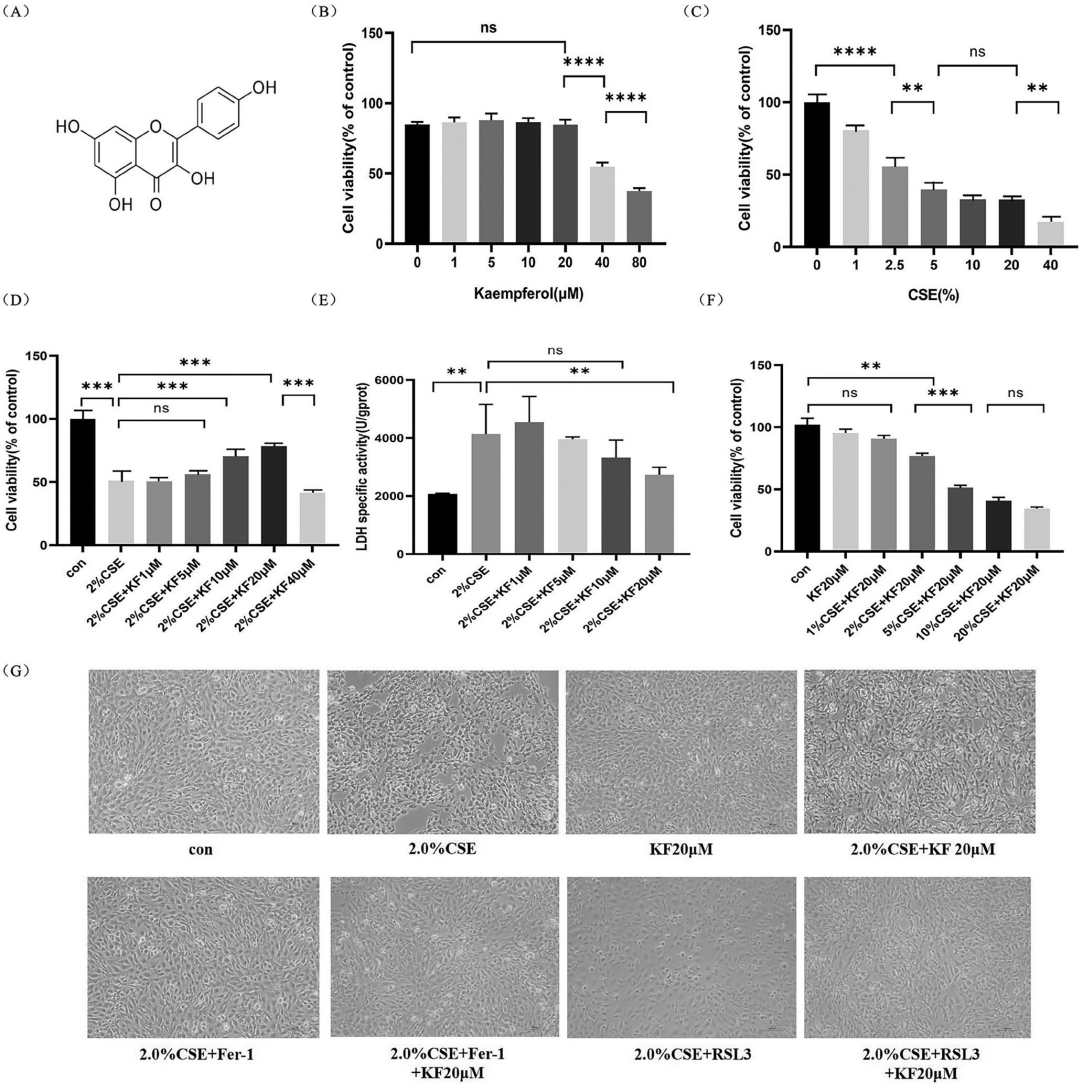
Kaempferol enhances cell viability and attenuates cytotoxicity in CSE‐exposed BEAS‐2B cells. (A) Chemical structure of kaempferol. (B) MTS assay results showing the inhibition rate of BEAS‐2B cell proliferation after co‐incubation with kaempferol at concentrations of 0, 1, 5, 10, 20, 40 and 80 μM for 24 h. (C) MTS assay evaluation of CSE‐induced cytotoxicity at increasing concentrations of 0%, 1%, 2.5%, 5%, 10%, 20% and 40% for 24 h. (D‐E) Assessment of (by MTS assay) and cytotoxicity (by LDH assay) in BEAS‐2B cells pre‐treated with kaempferol (1, 5, 10, 20 μM) for 1 h prior to 24‐h exposure to 2% CSE. (F) MTS assay measurement of cell viability in BEAS‐2B cells pretreated with 20 μM kaempferol for 1 h and subsequently exposed to various concentrations of CSE (1%, 2%, 5%, 10%, 20%) for 24 h. (G) Representative images depicting morphological changes in each treatment group. All experiments were performed in triplicate, and data are presented as mean ± SD. Statistical significance is indicated as *p < 0.05, **p < 0.01, ***p < 0.001, ****p < 0.0001 compared to the control group. Error bars represent the standard deviation (SD).

The chemical structure of kaempferol (KF) is shown in Figure 1A. To evaluate its effect on cell viability, BEAS‐2B cells were incubated with various concentrations of kaempferol (1–80 μM) for 24 h, followed by an MTS assay. Kaempferol exhibited no cytotoxicity at concentrations of 1–20 μM; however, at higher concentrations (40–80 μM), significant inhibitory effects on cell viability were observed (*p* < 0.0001) (Figure 1B). Next, to evaluate the effect of CSE on cell viability, BEAS‐2B cells were exposed to different concentrations of CSE (1%–40%) for 24 h. The MTS assay results demonstrated a dose‐dependent increase in cytotoxicity (Figure 1C). To identify the optimal protective concentration of kaempferol against CSE‐induced cytotoxicity, BEAS‐2B cells were pretreated with kaempferol (1–40 μM) for 1 h and subsequently exposed to 2% CSE for 24 h. Treatment with 2% CSE alone resulted in approximately 50% cytotoxicity (*p* < 0.001). However, pretreatment with 1 μM, 5 μM or 40 μM kaempferol did not significantly reduce CSE‐induced cytotoxicity (Figure 1D,E). In contrast, pre‐treatment with 10 μM and 20 μM kaempferol significantly reduced cytotoxicity and improved cell viability (*p* < 0.01) (Figure 1E,G).

To further investigate the protective effects of kaempferol, BEAS‐2B cells were pretreated with 20 μM kaempferol for 1 h and then exposed to varying concentrations of CSE (1%, 2%, 5%, 10%, 20%) for 24 h. Although the combination of KF with 5%–20% CSE still led to substantial cytotoxicity (> 50%, *p* < 0.01), overall cell viability remained relatively stable in the 2% CSE + 20 μM KF group (Figure 1F). Collectively, these results indicate that kaempferol at concentrations of 10 μM and 20 μM confers significant protection against 2% CSE‐induced cytotoxicity in BEAS‐2B cells, with 20 μM offering the most pronounced protective effect.

In supplementary experiments, we also examined how other lung cell lines responded to CSE and kaempferol. H1299 cells showed marked resistance to CSE, maintaining a survival rate of 90.14% ± 5.7% after 24 h of treatment with 10% CSE (Figure S1). In contrast, A549 cells were highly sensitive to 5% CSE, displaying only 41.1% ± 2.6% viability (Figure S2). Notably, A549 cells harbor a KRAS mutation that is associated with constitutive Nrf2 activation [6]. This elevated basal antioxidant activity may reduce the incremental benefit of kaempferol, resulting in only a modest increase in cell survival (55.15% ± 3.1%, *p* = 0.74) upon treatment with 20 μM kaempferol (Figure S3). These findings underscore the cell line–specific responses to CSE and highlight that, while kaempferol effectively protects BEAS‐2B cells from moderate CSE exposure, its efficacy may be limited in certain genetic contexts.

### 3.2 Kaempferol Ameliorates CSE‐Induced Ferroptosis‐Related Changes in BEAS‐2B Cells

**FIGURE 2:**
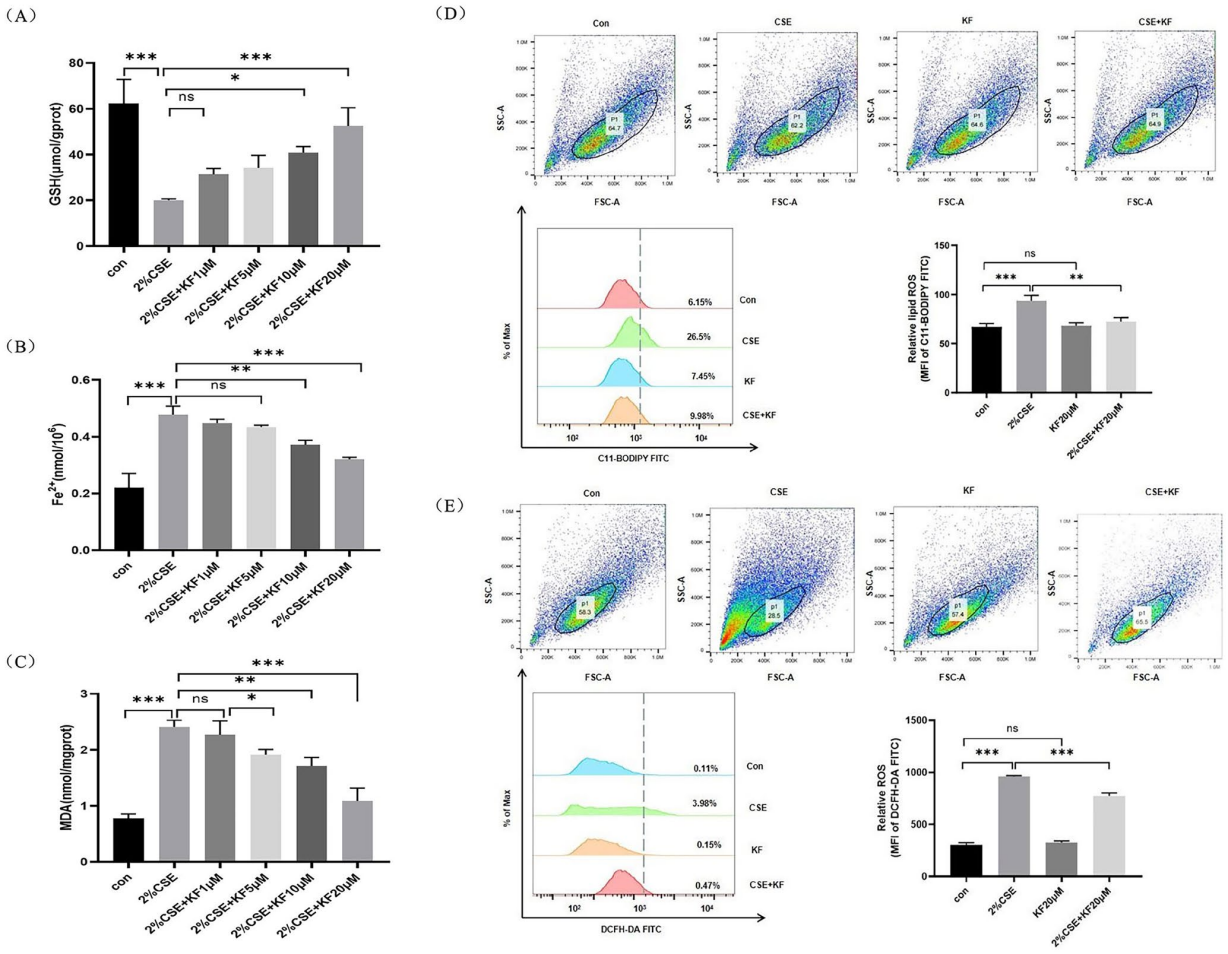
Kaempferol ameliorates CSE‐induced ferroptosis‐related changes in BEAS‐2B cells. (A) Quantification of cellular GSH levels, (B) measurement of intracellular ferrous iron (Fe^2+^) content, (C) determination of malondialdehyde (MDA) levels in BEAS‐2B cells treated with kaempferol (1, 5, 10, 20 μM) in the presence of 2% CSE using appropriate colorimetric assays. Cells were pre‐treated with 20 μM kaempferol for either 1 or 24 h prior to CSE exposure. (D) Lipid peroxidation ROS was assessed via C11‐BODIPY staining, and (E) intracellular ROS levels were measured using DCFH‐DA staining, with flow cytometric analysis. Data are representative of three independent experiments and expressed as mean ± SD. Statistical significance is indicated as *p < 0.05, **p < 0.01, ***p < 0.001, ****p < 0.0001, compared with the control group. Error bars represent standard deviation (SD).

To evaluate the effect of kaempferol on CSE‐induced ferroptosis‐related metabolic changes in BEAS‐2B cells, the levels of GSH, intracellular Fe^2+^, and malondialdehyde (MDA) were measured. Exposure to 2% CSE significantly decreased GSH levels, while increasing Fe^2+^ and MDA levels compared to control groups (Figure 2A–2C). Pre‐treatment with kaempferol (10 μM and 20 μM) markedly reversed the CSE‐induced decrease in GSH (*p* < 0.05) and significantly reduced the elevated Fe^2+^ and MDA levels (*p* < 0.01). In addition, analysis via flow cytometry using C11‐BODIPY and DCFH‐DA probes revealed that 2% CSE markedly increased lipid peroxidation and intracellular ROS levels (*p* < 0.01), whereas kaempferol (20 μM) pre‐treatment significantly attenuated these ROS elevations (Figure 2D,E). These results suggest that kaempferol effectively mitigates CSE‐induced ferroptosis‐related changes in BEAS‐2B cells by modulating metabolic markers and reducing ROS accumulation.

### 3.3 Kaempferol Attenuates CSE‐Induced Ferritinophagy in BEAS‐2B Cells

**FIGURE 3:**
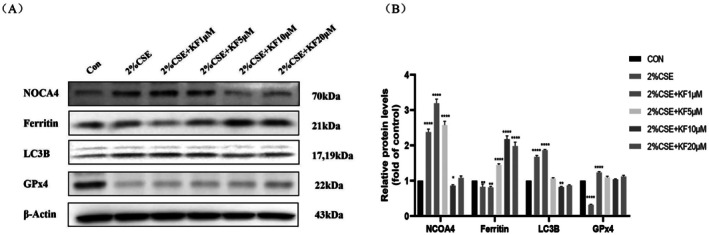
Kaempferol attenuates CSE‐induced ferritinophagy in BEAS‐2B cells. BEAS‐2B cells were pretreated with kaempferol at concentrations of 1, 5, 10, 20 μM for 1 h, followed by 24‐h stimulation with 2% CSE. (A) Western blot analysis was performed to assess protein levels of NCOA4, ferritin, LC3B, and GPx4. (B) Semi‐quantitative analysis of above proteins. Data are representative of three independent experiments and are expressed as mean ± SD. Statistical significance is indicated as *p < 0.05, **p < 0.01, ***p < 0.001, ****p < 0.0001, compared with the control group. Error bars represent standard deviation (SD).

Ferritin autophagy, a key process in the degradation of ferritin, plays a crucial role in the accumulation of labile iron and the promotion of ferroptosis. CSE triggers NCOA4‐mediated ferritinophagy, leading to increased intracellular free iron accumulation, which contributes to ferroptosis in the pathogenesis of COPD. To investigate the effect of kaempferol on CSE‐induced ferritinophagy in BEAS‐2B cells, we pre‐treated the cells with increasing concentrations of kaempferol (1, 5, 10, 20 μM) for 1 h, followed by stimulation with 2% CSE for 24 h. Western blot analysis revealed that 2% CSE treatment significantly downregulated ferritin and GPx4 protein levels (*p* < 0.05), with reductions of 17% and 68%, respectively. In contrast, kaempferol treatment restored ferritin and GPx4 levels in a dose‐dependent manner; notably, 20 μM kaempferol increased ferritin and GPx4 levels by 115% and 80%, respectively (*p* < 0.05). Additionally, CSE‐induced elevations in NCOA4 and LC3B (increased by 137% and 67%, respectively; *p* < 0.01) were dose‐dependently suppressed by kaempferol, with 20 μM reducing NCOA4 and LC3B by 129% and 80%, respectively (*p* < 0.01) (Figure 3A,B). These results suggest that kaempferol may alleviate CSE‐induced ferritinophagy in BEAS‐2B cells.

### 3.4 Kaempferol Attenuates CSE‐Induced Ferroptosis by Modulating GPx4 in BEAS‐2B Cells

**FIGURE 4:**
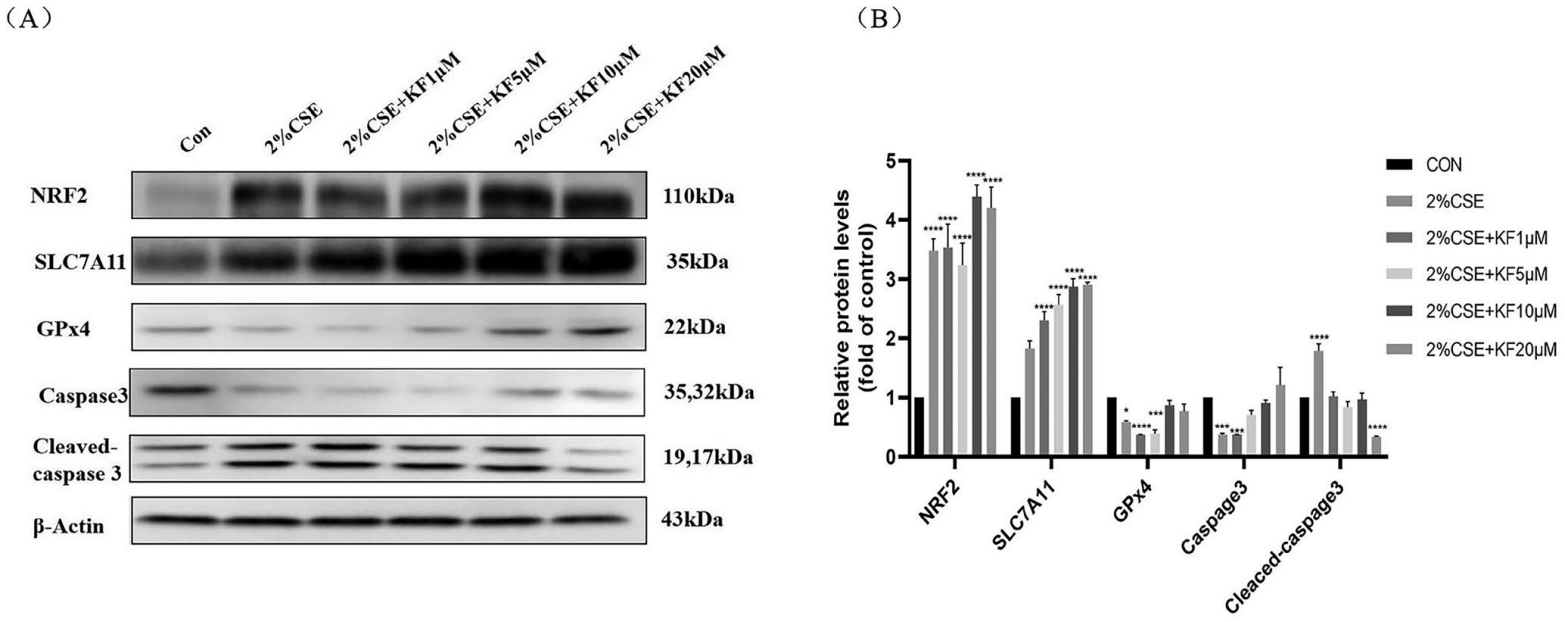
Kaempferol attenuates CSE‐induced ferroptosis in BEAS‐2B cells. BEAS‐2B cells were pretreated with kaempferol at concentrations of 1, 5, 10, 20 μM for 1 h, followed by 24‐h stimulation with 2% CSE. (A) Western blot evaluation of Nrf2, SLC7A11, GPx4, caspase 3, and cleaved‐caspase 3. (B) Corresponding semi‐quantitative analysis. Data are representative of three independent experiments and are expressed as mean ± SD. Statistical significance is indicated as *p < 0.05, **p < 0.01, ***p < 0.001, ****p < 0.0001, compared with the control group. Error bars represent standard deviation (SD).

GPx4 is a central regulatory enzyme in ferroptosis, acting to reduce lipid peroxidation and thus negatively regulating ferroptosis. Previous studies have demonstrated that CSE‐induced ferroptosis primarily arises from the depletion of GPx4. To examine whether kaempferol protects BEAS‐2B cells from CSE‐induced ferroptosis by preserving GPx4, we pretreated cells with kaempferol (1, 5, 10 or 20 μM) for 1 h before exposing them to 2% CSE for 24 h. Western blot analysis revealed that kaempferol restored the protein levels of GPx4 and caspase‐3, both of which were downregulated by CSE. Notably, the cleaved form of caspase‐3, which was significantly elevated in the 2% CSE‐only group (*p* < 0.01), decreased in a dose‐dependent manner with kaempferol pretreatment (*p* < 0.01). For instance, 20 μM kaempferol reduced the level of cleaved caspase‐3 by approximately 45% compared with the 2% CSE‐treated group. Furthermore, NRF2 and SLC7A11 were significantly upregulated by 2% CSE compared with the control group (*p* < 0.01), showing increases of 48% and 82%, respectively. This upregulation was further enhanced by kaempferol in the 20 μM kaempferol + CSE group; NRF2 and SLC7A11 levels were elevated by 71% and 108%, respectively, compared with the CSE‐only group (Figure 4A,B). Collectively, these findings suggest that kaempferol attenuates CSE‐induced ferroptosis by modulating GPx4, NRF2 and SLC7A11, highlighting its potential therapeutic value in mitigating CSE‐induced cellular damage.

### 3.5 Kaempferol Modulates Nrf2/NCOA4/GPx4 Axis to Alleviate CSE‐Induced Ferritinophagy and Ferroptosis in BEAS‐2B Cells

**FIGURE 5:**
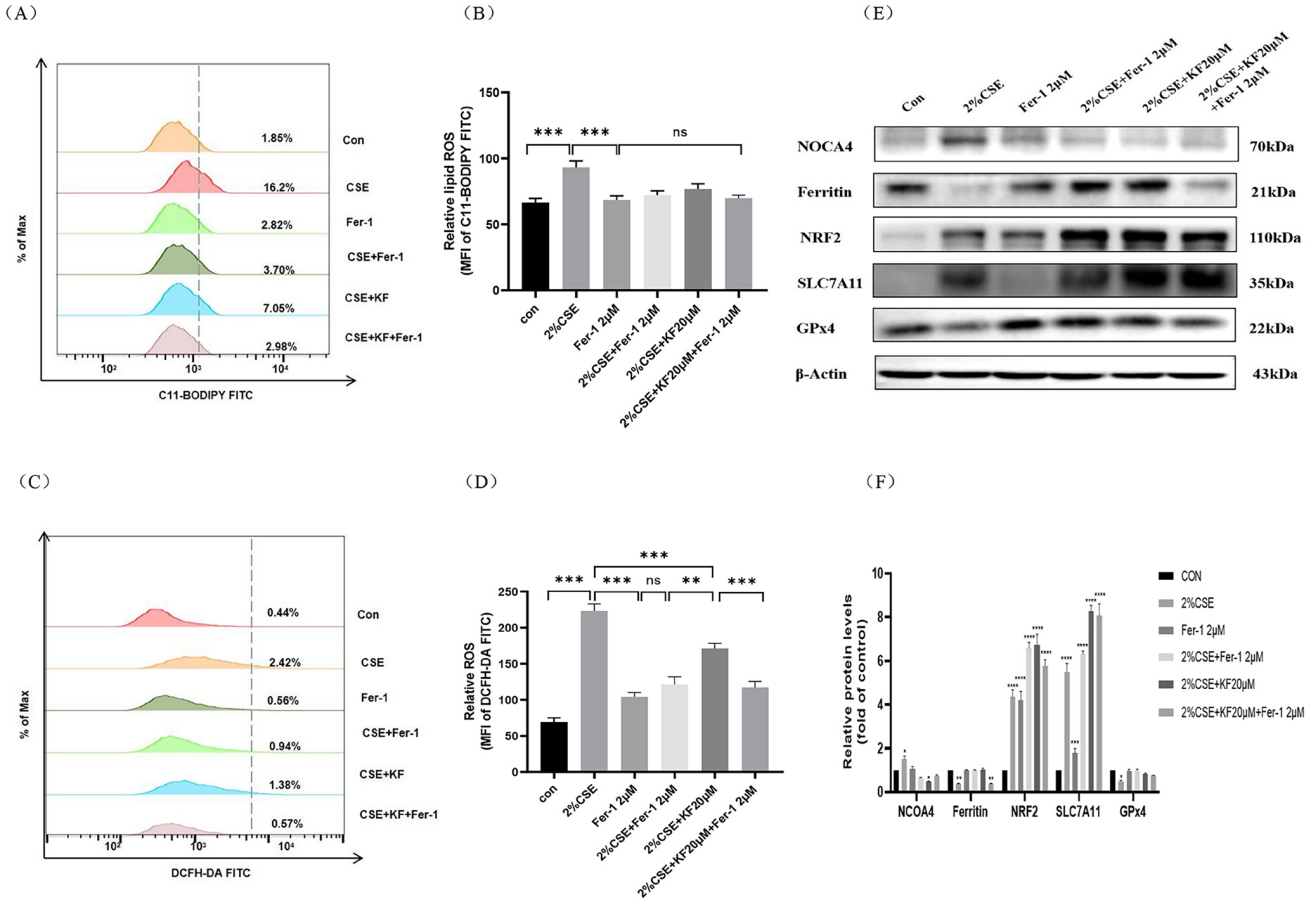
Kaempferol enhances the inhibitory effect of Fer‐1 on CSE‐induced ferritinophagy and ferroptosis through modulation of the Nrf2/NCOA4/GPx4 axis. BEAS‐2B cells were pretreated with 20 μM kaempferol alone or in combination with 2 μM Fer‐1 for 1 h, followed by stimulation with 2% CSE for either 16 or 24 h. (A, B) Lipid peroxidant ROS levels were assessed using C11‐BODIPY staining, (C, D) intracellular ROS levels were measured using DCFH‐DA staining and analysed by flow cytometry. (E) Western blot analysis determined protein expression levels of NCOA4, ferritin, Nrf2, SLC7A11, GPx4. (F) Semi‐quantitative analysis provided for protein bands. Data are representative of three independent experiments and are expressed as mean ± SD. Statistical significance is indicated as *p < 0.05, **p < 0.01, ***p < 0.001, ****p < 0.0001, compared with the control group. Error bars represent standard deviation (SD).

Nrf2 is a critical transcription factor known to mitigate ferroptosis by regulating key ferroptosis‐associated proteins, including SLC7A11, GPx4, and ferritin [3, 6, 12, 13, 14]. To investigate the protective mechanism of kaempferol, BEAS‐2B cells were pretreated with 20 μM kaempferol alone or in combination with 2 μM Fer‐1 (a ferroptosis inhibitor) or 5 μM RSL3 (a GPx4 inhibitor and ferroptosis agonist) for 1 h. The cells were then exposed to 2% CSE for 16 or 24 h, followed by flow cytometry and Western blot analyses. Flow cytometry revealed that 2% CSE significantly elevated both lipid peroxidation and intracellular ROS levels compared to the control (*p* < 0.01). Pretreatment with kaempferol or Fer‐1, whether alone or combined, markedly reduced ROS levels, with the most pronounced decrease observed in the 2% CSE + kaempferol + Fer‐1 group (Figure 5A–D). Western blot results revealed that 2% CSE upregulated NCOA4, Nrf2, and SLC7A11 (*p* < 0.05) while downregulating ferritin and GPx4 (*p* < 0.05). Co‐treatment with kaempferol or Fer‐1 reversed these effects by reducing NCOA4 levels and restoring ferritin, Nrf2, SLC7A11 and GPx4 expression relative to the 2% CSE‐only group (Figure 5E,F).

**FIGURE 6:**
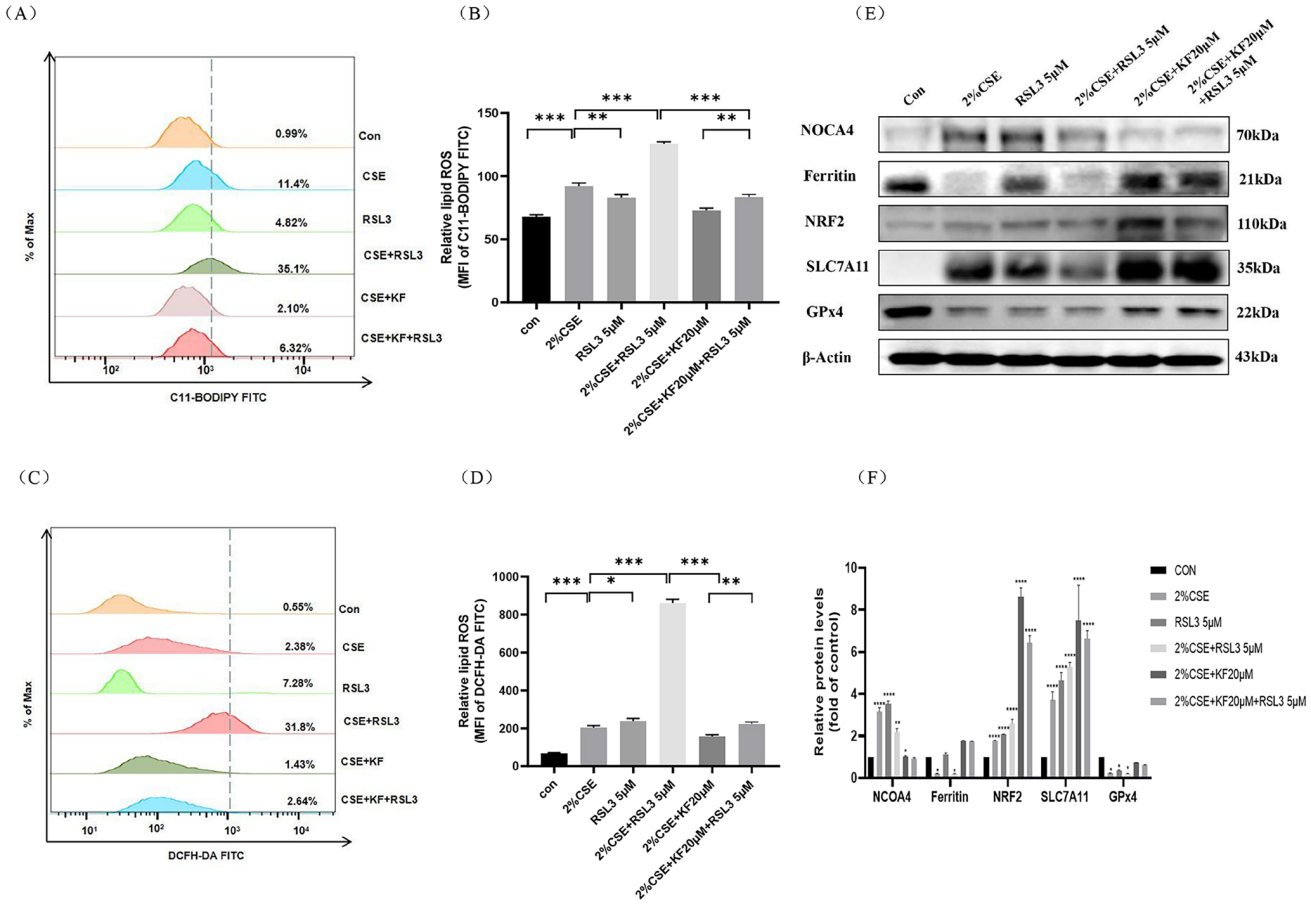
Kaempferol reverses the accelerating effect of RSL3 on CSE‐induced ferritinophagy and ferroptosis by modulating the Nrf2/NCOA4/GPx4 axis. BEAS‐2B cells were pretreated with 20 μM kaempferol alone or in combination with 5 μM RSL3 for 1 h, followed by stimulation with 2% CSE for 16 or 24 h. (A, B) Lipid peroxidant ROS levels determined using C11‐BODIPY staining, (C, D) intracellular ROS levels were measured with DCFH‐DA staining and analysed by flow cytometry. (E) Protein expression levels of NCOA4, ferritin, Nrf2, SLC7A11, and GPx4 were evaluated by Western blot. (F) Semi‐quantitative analysis provided for protein bands. Data are representative of three independent experiments and are expressed as mean ± SD. Statistical significance is indicated as *p < 0.05, **p < 0.01, ***p < 0.001, ****p < 0.0001, compared with the control group. Error bars represent standard deviation (SD).

To assess the impact of GPx4 inhibition, cells were treated with RSL3 in addition to 2% CSE. The 2% CSE + RSL3 group showed a further increase in both lipid peroxidation and intracellular ROS (*p* < 0.01) compared to 2% CSE alone. However, when kaempferol was added (2% CSE + RSL3 + kaempferol), ROS levels were significantly reduced, demonstrating kaempferol's capacity to mitigate oxidative stress even under GPx4‐inhibited conditions (Figures 6A–D). Western blot data confirmed that RSL3 further suppressed ferritin and GPx4 while upregulating NCOA4, Nrf2 and SLC7A11 expression (*p* < 0.05). Notably, kaempferol co‐treatment reversed these alterations by significantly reducing NCOA4 and increasing ferritin, Nrf2, SLC7A11 and GPx4 levels compared to both the 2% CSE and 2% CSE + RSL3 groups (Figure 6E,F).

Overall, these findings demonstrate that kaempferol alleviates CSE‐induced ferritinophagy and ferroptosis in BEAS‐2B cells by modulating the Nrf2/NCOA4/GPx4 axis. By reducing ROS accumulation and restoring the expression of key ferroptosis‐related proteins, kaempferol shows promise as a protective agent against CSE‐induced cellular damage.

## 4 Discussion

Cigarette smoke contains numerous toxic compounds that elevate intracellular reactive oxygen species (ROS) and compromise antioxidant defences, thereby playing a pivotal role in the development of chronic obstructive pulmonary disease (COPD) [13, 15]. While conventional therapies, such as antibiotics and bronchodilators, alleviate respiratory symptoms, they fail to prevent disease progression. Consequently, identifying novel therapeutic targets is critical. In recent years, ferroptosis—an iron‐dependent, regulated cell death mechanism—has emerged as a key contributor to cigarette smoke extract (CSE)‐induced lung injury [16].

In the current study, we investigated the protective effects of kaempferol, a bioactive flavonoid, on CSE‐induced injury in BEAS‐2B airway epithelial cells. MTS assays demonstrated that kaempferol at optimal concentrations (10 μM and 20 μM) significantly restored cell viability and reduced cytotoxicity induced by 2% CSE, whereas lower or excessively high concentrations were less effective. These results suggest that kaempferol exerts a dose‐dependent protective effect against CSE‐induced cellular damage.

Ferroptosis is characterised by an imbalance between oxidative damage and antioxidant defences, with dysregulated iron metabolism playing a central role [6, 7]. Under normal conditions, iron homeostasis is tightly maintained; however, CSE disrupts this balance by increasing the labile iron pool and enhancing lipid peroxidation [17]. Our data indicate that kaempferol restores redox homeostasis by elevating glutathione (GSH) levels and reducing the accumulation of malondialdehyde (MDA) and Fe^2+^, thereby both scavenging ROS and regulating iron metabolism.

Mechanistically, kaempferol modulates the Nrf2/NCOA4/GPx4 signalling axis—a central pathway in cellular antioxidant defence. Under oxidative stress, Nrf2 translocates to the nucleus and upregulates antioxidant enzymes, including SLC7A11 and GPx4 [6, 12, 14]. We observed that CSE exposure induced a compensatory upregulation of Nrf2, which was further enhanced by kaempferol treatment. This activation correlated with increased GPx4 expression, a key enzyme in detoxifying lipid peroxides, and with a concomitant reduction in nuclear receptor coactivator 4 (NCOA4) expression, thereby preserving ferritin levels and suppressing ferritinophagy.

Recent studies have demonstrated that CSE triggers NCOA4‐mediated selective autophagy of ferritin (ferritinophagy), which disrupts intracellular iron homeostasis and promotes ferroptosis, contributing to persistent lung injury [18, 19]. Ferritin plays a critical role in the iron regulatory network by sequestering Fe^2+^ and storing it as Fe^3+^; under iron‐deficient conditions, ferritin releases stored iron via an autophagy‐lysosomal pathway mediated by LC3 [20]. The autophagic degradation of ferritin disturbs iron homeostasis, triggers the Fenton reaction and increases oxidative stress [21] [22, 23, 24]. For instance, in A549 cells, CSE initially increases intracellular ferritin levels, which then gradually decline with prolonged exposure—suggesting mobilisation of ferritin for iron utilisation [25]. Furthermore, GPx4, a selenoprotein that directly reduces peroxidised phospholipids in the plasma membrane [26], serves as the central regulatory enzyme in ferroptosis; its deficiency in mice leads to ferroptosis in renal tubular cells and acute kidney injury [27].

**FIGURE 7:**
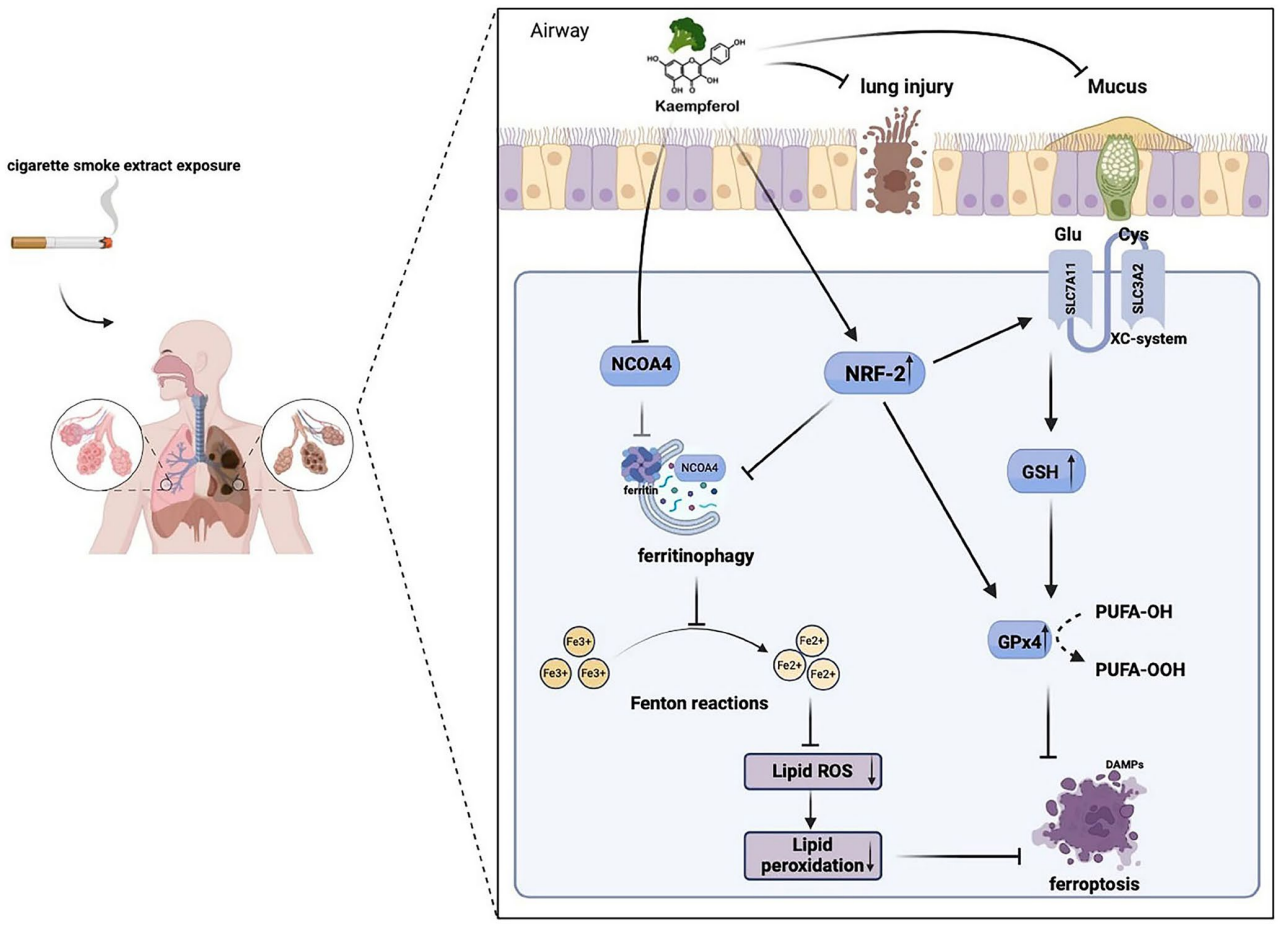
Schematic model illustrating the mechanistic pathway of Kaempferol in mitigating CSE‐induced lung injury and ferroptosis. Kaempferol modulates the Nrf2/NCOA4/GPx4 axis in airway epithelial cells by enhancing Nrf2 activation to upregulate GPx4 expression and suppressing NCOA4‐mediated ferritinophagy. This dual action restricts free iron release, reduces lipid peroxidation, and restores redox homeostasis, ultimately preventing ferroptotic cell death.

In our study, exposure of the human bronchial epithelial BEAS‐2B cells to CSE markedly increased Nrf2 expression, likely as a compensatory response to oxidative stress. Kaempferol further enhanced Nrf2 activation, thereby preventing CSE‐induced ferroptosis. As illustrated in Figure 7, CSE triggers NCOA4‐mediated ferritinophagy, releasing Fe^2+^ that drives lipid peroxidation through the Fenton reaction, ultimately culminating in ferroptosis. By enhancing Nrf2 activity, kaempferol increases GPx4 expression and preserves ferritin levels, effectively limiting free iron release and mitigating oxidative damage [28]. The ferroptosis inhibitor Fer‐1 and the GPx4 inhibitor/ferroptosis inducer RSL3 confirmed that kaempferol's protective effects are mediated through the Nrf2/NCOA4/GPx4 axis. These findings underscore kaempferol's potential to restore redox balance, reduce lipid ROS, and protect against ferroptotic cell death, thereby offering a promising natural therapeutic strategy for managing CSE‐induced lung injury in COPD.

Collectively, our findings demonstrate that kaempferol exerts a multifaceted protective effect against CSE‐induced lung injury by restoring redox homeostasis and modulating the Nrf2/NCOA4/GPx4 signalling axis. These insights advance our understanding of COPD pathogenesis and support the potential application of kaempferol as a natural therapeutic agent for this disease.

## Author Contributions

**Fengri Jin:** data curation (equal), methodology (lead), writing – original draft (equal), writing – review and editing (equal). **Yanfei Xing:** writing – original draft (equal). **Songyu Li:** writing – original draft (equal). **Zhennan Yi:** writing – original draft (equal). **Aibin Wu:** writing – original draft (equal). **Xin Li:** writing – original draft (equal), writing – review and editing (equal).

## Funding

This work was supported by the Competitive Allocation Project of the Zhanjiang Municipal Science and Technology Development Special Fund (2021A05124).

## Ethics Statement

The authors have nothing to report.

## Consent

All authors consent to the publication of this.

## Conflicts of Interest

The authors declare no conflicts of interest.

## Supporting information


**Figure S1:** Cytotoxicity effect of CSE on H1299 cells. H1299 cells were exposed to increasing concentrations CSE (0%, 1%, 2.5%, 5%, 10%, 20% and 40%) for 24 h, and cytotoxicity was assessed using the MTS assay. All experiments were performed in triplicate, and data are presented as mean ± SD. Statistical significance is indicated as *p < 0.05, **p < 0.01, ***p < 0.001, ****p < 0.0001 compared to the control group. Error bars represent the standard deviation (SD).

**Figure S2:** Cytotoxicity effect of CSE on A549 cells. A549 cells were treated with increasing concentrations of CSE (0%, 1%, 2.5%, 5%, 10%, 20% and 40%) for 24 h, and the cytotoxicity was measured using the MTS assay. All experiments were performed in triplicate, and data are presented as mean ± SD. Statistical significance is indicated as *p < 0.05, **p < 0.01, ***p < 0.001, ****p < 0.0001 compared to the control group. Error bars represent the standard deviation (SD).

**Figure S3:** Cytotoxicity effect of Kaempferol on A549 cells pretreated with 5% CSE. A549 cells were pretreated with 5% CSE for 24 h, followed by treatment with Kaempferol at increasing concentrations for an additional 24 h. Cytotoxicity was evaluated using MTS assay. All experiments were performed in triplicate, and data are presented as mean ± SD. Statistical significance is indicated as *p < 0.05, **p < 0.01, ***p < 0.001, ****p < 0.0001 compared to the control group. Error bars represent the standard deviation (SD).

## Data Availability

The data supporting the conclusions of this study have been publicly stored in Figshare, and the website address is http://doi.org/10.6084/m9.figshare.30971053
