# Supplementary figures and images for: Kaempferol Mitigates CSE‐Induced Lung Injury and Epithelial Cell Ferroptosis via Modulating Nrf2/NCOA4/GPx4 Axis
Source: J Cell Mol Med. 2025 Dec 31;30(1):e71010. doi: 10.1111/jcmm.71010 (PMC12755056; doi:10.1111/jcmm.71010)

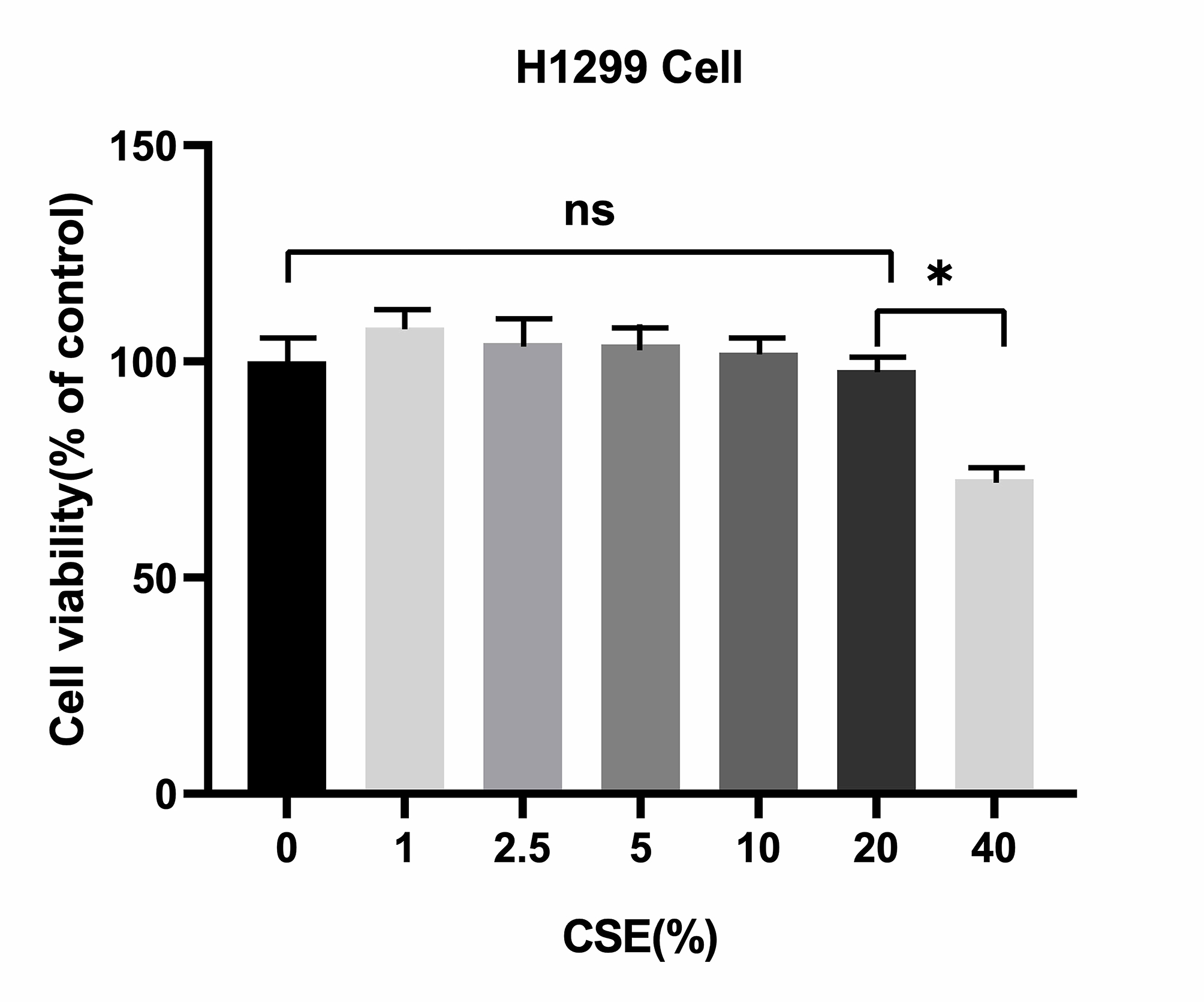

Supplement: Supplementary file 1 — Figure S1: Cytotoxicity effect of CSE on H1299 cells. H1299 cells were exposed to increasing concentrations CSE (0%, 1%, 2.5%, 5%, 10%, 20% and 40%) for 24 h, and cytotoxicity was assessed using the MTS assay. All experiments were performed in triplicate, and data are presented as mean ± SD. Statistical significance is indicated as *p < 0.05, **p < 0.01, ***p < 0.001, ****p < 0.0001 compared to the control group. Error bars represent the standard deviation (SD). [file JCMM-30-e71010-s003.jpg]

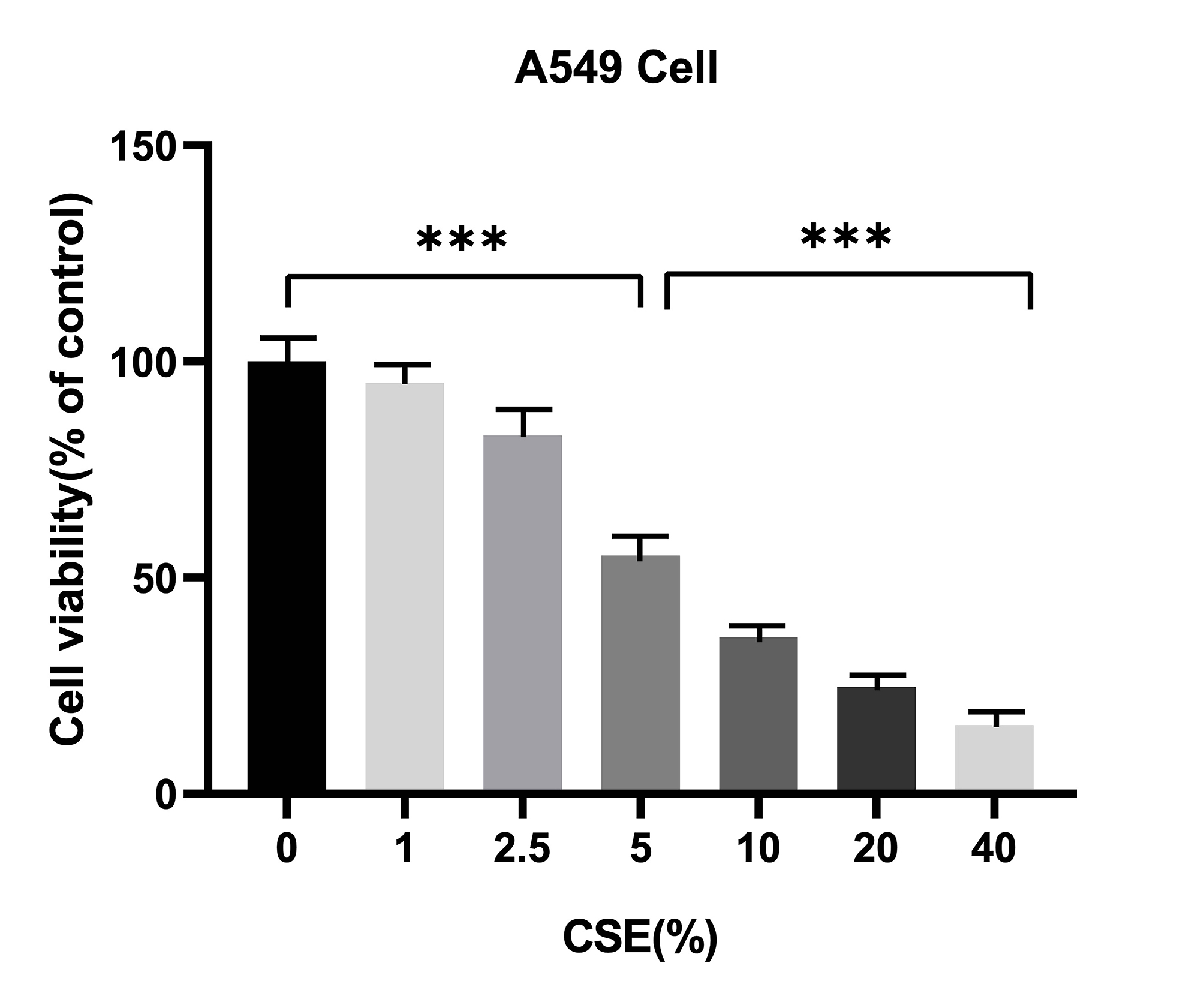

Supplement: Supplementary file 2 — Figure S2: Cytotoxicity effect of CSE on A549 cells. A549 cells were treated with increasing concentrations of CSE (0%, 1%, 2.5%, 5%, 10%, 20% and 40%) for 24 h, and the cytotoxicity was measured using the MTS assay. All experiments were performed in triplicate, and data are presented as mean ± SD. Statistical significance is indicated as *p < 0.05, **p < 0.01, ***p < 0.001, ****p < 0.0001 compared to the control group. Error bars represent the standard deviation (SD). [file JCMM-30-e71010-s001.jpg]

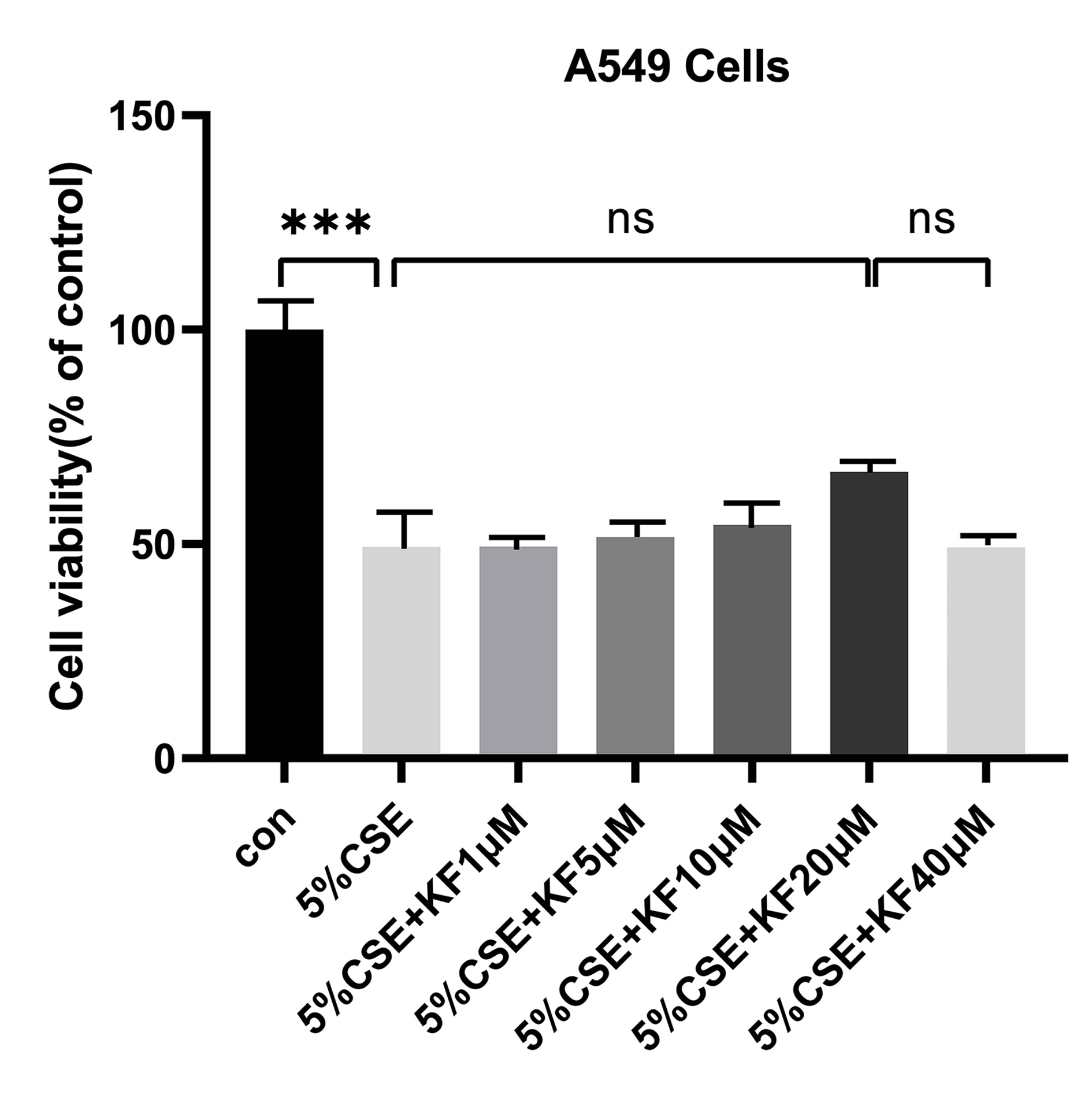

Supplement: Supplementary file 3 — Figure S3: Cytotoxicity effect of Kaempferol on A549 cells pretreated with 5% CSE. A549 cells were pretreated with 5% CSE for 24 h, followed by treatment with Kaempferol at increasing concentrations for an additional 24 h. Cytotoxicity was evaluated using MTS assay. All experiments were performed in triplicate, and data are presented as mean ± SD. Statistical significance is indicated as *p < 0.05, **p < 0.01, ***p < 0.001, ****p < 0.0001 compared to the control group. Error bars represent the standard deviation (SD). [file JCMM-30-e71010-s002.docx]
